# Temperature Control Method for Electric Heating Furnaces Based on Auto-Encoder and Fuzzy PI Control

**DOI:** 10.3390/s25165020

**Published:** 2025-08-13

**Authors:** Haiyang Huang, Yingmao Luo, Chun Zhao, Hui Suo

**Affiliations:** State Key Laboratory of Integrated Optoelectronics, College of Electronic Science and Engineering, Jilin University, Changchun 130012, China; huanghy24@mails.jlu.edu.cn (H.H.); luoym24@mails.jlu.edu.cn (Y.L.); zchun@jlu.edu.cn (C.Z.)

**Keywords:** electric heating furnace, temperature control, dynamic modelling, auto-encoder, fuzzy control

## Abstract

**Highlights:**

Aiming at the difficult problem of controlling the electric heating furnace, combining the advantages of Auto-Encoder and fuzzy control, a composite control algorithm is proposed for dynamic modelling of the electric heating furnace, predicting the future temperature and adjusting the control parameters in real time in order to achieve accurate and stable control of the temperature, which provides a new way of thinking for solving the control problems of nonlinear, time-varying, and large-time-lag systems.

**What are the main findings?**

- A discrete mathematical model of an electric heating furnace was established, and unsupervised dynamic modelling was achieved through an auto-encoder.
- A control structure combining predictive compensation and fuzzy regulation was designed to achieve stable low-overshoot control under complex interference.

**What is the implication of the main finding?**

- Improved the stability, accuracy, and robustness of the electric heating furnace temperature control system.
- Provided a new modelling and control framework for solving control problems in nonlinear, time-varying, and large-time-delay industrial heating systems, with good application prospects.

**Abstract:**

Electric heating furnaces are widely used in industrial production and scientific research, where the quality of temperature control directly affects product performance and operational safety. However, precise control remains challenging due to the system’s nonlinear behaviour, time-varying characteristics, and significant time delays. To overcome these issues, this paper proposes a composite control method that integrates an auto-encoder-based prediction model with fuzzy PI control. Specifically, a discrete-time temperature model is constructed, in which the auto-encoder learns the system dynamics and predicts future temperatures, while the fuzzy controller adaptively tunes the PI parameters in real time. This approach improves both modelling accuracy and the adaptability of the control system. The simulation results on the MATLAB/Simulink platform show that the proposed method maintains the temperature overshoot within 2% under various disturbances, including a maximum delay of 243 s, ±2 °C measurement noise, 10% voltage fluctuation, and abrupt 10% gain variation. These results demonstrate the method’s strong robustness and indicate its suitability for advanced control design in complex industrial environments.

## 1. Introduction

The electric heating furnace is widely used in industrial and research fields such as material sintering, gas reactions [1], metal heat treatment [2], and integrated circuit manufacturing [3] due to its advantages of rapid temperature rise, pollution-free operation, and flexible control. The stability and accuracy of temperature regulation have a direct impact on product quality and production safety. Improper temperature control may result in substandard product performance, material damage, increased energy consumption, or even equipment failures or safety incidents [4]. As a typical intelligent sensing system, the electric heating furnace collects temperature information from the front-end sensors, and the back-end control system processes and models the sensed data and outputs heating commands, which constitutes a closed-loop structure of ‘sensing–decision making–execution’. As the middle and back end of the sensing link, the performance of the control system directly determines whether the sensed data can be efficiently converted into a stable thermal regulation response. Therefore, achieving stable, precise, and rapid-response temperature control in electric heating furnaces is of great significance.

However, the electric heating process is characterized by nonlinearity, time-variation, and large time lag, which makes it difficult to obtain satisfactory control results with conventional PID control [5,6]. Especially in systems with significant time delays, PID control is prone to overshooting and oscillation, which makes it difficult to meet the high-performance control demand of accurate, stable, and fast response [7]. Meanwhile, the optimal PID parameters need to be rectified in advance, which not only increases the time overhead of control algorithm deployment [8], but also leads to the control effect depending on the model accuracy.

To address the above problems, researchers have proposed many improvement methods. Among them, one class of methods is dedicated to improving the adaptive ability and intelligence of the controller itself. Fuzzy control has been widely used in various types of temperature control systems [9,10] and nonlinear jump systems [11,12] because it does not need to rely on an accurate mathematical model, has good nonlinear processing capability, and has the property of real-time adjustment using expert experience [13,14]. Many researchers have combined fuzzy control with PID to improve the adaptability and robustness of the system by adjusting the control parameters in real time [15,16,17,18,19]. Zhou introduced an RBF neural network based on fuzzy PID to learn the nonlinear characteristics of the system and increase the adaptability of the controller [20]. Rajput et al. proposed a fuzzy PID controller optimized by a modified electric eel foraging algorithm, which improves the dynamic response and steady-state accuracy of electric furnace temperature control through intelligent parameter tuning [21]. Chen et al. proposed an adaptive control strategy based on the neural network for systems with non-strict feedback structure, which further extends the scope of application of neural network control in complex nonlinear systems [22]. Zhu combined PID with an improved Smith’s prediction compensation and differential tracker to achieve effective compensation of control signal lag [23]. Xu used both a fuzzy controller and Smith’s prediction compensation to reduce the overshoot to 3% (250 °C) in a system with a delay of 95 s [1]. Karan and Dey, on the other hand, designed a Smith predictor based on an IMC structure for a large-lag system, which effectively improves the control accuracy of a system with a right half-plane zero point [24].

Another class of research focuses on improving the modelling accuracy and strengthening the ability to perceive the dynamics of the system. Zhou and Luo modelled the ramp-up and ramp-down states of an electric heating furnace separately and used a genetic algorithm to automatically tune the PID parameters [25]. Lusenko established three discrete models of the system function of a waste-heat boiler and used different models at different operating temperatures, which significantly improved the closed-loop response performance of the system [26]. Lan et al. combined the generalized predictive control with BP neural network PI control to achieve fast response and stable control of temperature in complex environments [27]; Chen et al. proposed an observer-based neural network control and fuzzy PID algorithm optimized by a genetic algorithm, respectively, which effectively enhanced the system’s nonlinear modelling capability [22,28]. Pergantis et al. developed a predictive control framework that integrates thermal modelling, disturbance forecasting, and convex MPC to dynamically adjust set-points for balancing energy efficiency and responsiveness under strong nonlinearity and long delays [29].

In terms of engineering implementation, the goal of a temperature control system is not only the control accuracy, but also the response speed, robustness, and anti-interference ability. In order to improve the system robustness and global optimization capability, some studies have introduced swarm intelligent optimization algorithms. Charkoutsis et al. used particle swarm algorithms to tune a nonlinear PID and improve its dynamic performance and steady-state error [30]. Liao et al. designed a hybrid PSO-fuzzy neural network structure for the decoupling control of a heating furnace, which effectively improved the decoupling capability and dynamic robustness of the system [31]. Tang et al. proposed a MIMO control algorithm integrating Smith prediction, fuzzy immune regulation, and PID structure, which showed excellent stability and robustness in the network control system [32].

Although the aforementioned studies have made significant progress in controller structure optimization, modelling accuracy improvement, and the introduction of intelligent algorithms, two critical issues remain inadequately addressed. First, existing thermal process models for electric heating furnaces are predominantly based on first-order inertial delay systems or their combinations. While effective in specific scenarios, these models lack a unified (i.e., a physically meaningful, non-composite model structure that remains consistent across different operating regimes), generalizable, and accurate formulation capable of adapting to varying furnace types and operating conditions. Second, the control performance heavily depends on model accuracy. Model-based feedforward compensation methods, such as Smith predictors, are particularly sensitive to modelling errors, which can lead to increased overshoot, slower response, or even control instability. These challenges are especially pronounced in electric heating systems characterized by large time delays, nonlinearity, and frequent disturbances, posing a substantial barrier to the practical deployment of high-performance control strategies.

This study aims to address the key issues constraining high-performance temperature control in electric heating furnaces by proposing a composite control method that integrates dynamic modelling with intelligent control. By combining auto-encoder-based system identification, delay-compensated prediction, and fuzzy PI parameter tuning, the proposed method forms a unified framework that reduces model dependency and enhances adaptability. Compared to traditional single-strategy approaches, this composite control method achieves lower overshoot, faster response, and stronger robustness under nonlinear and time-delayed conditions.

## 2. Materials and Methods

### 2.1. Establishment and Identification of Electric Heating Furnace Models

The effectiveness of temperature control is determined by the accuracy of the electric heating furnace model, so it is very important to establish an accurate and reliable model. This chapter establishes a discrete mathematical model of the electric heating furnace system and provides an automatic identification method for model parameters, which provides a theoretical basis for the design of control algorithms.

#### 2.1.1. Model Establishment

An electric furnace is a complex thermodynamic system, with furnace temperature influenced by factors such as heater heating, heat conduction through the furnace body and environment, convection at the heating surface, and radiation from external sources. Considering that the furnace is typically made of metal with high thermal conductivity and enclosed by effective insulation, radiative heat loss is minimal, and the vacuum or low-flow environment significantly weakens convective effects. Thus, consistent with the experimental findings in [33], dominant external heat dissipation occurs through conduction. Therefore, the overall temperature evolution of the furnace is mainly governed by the heating input and conductive losses to the surroundings.

The furnace temperature is sampled at a fixed frequency. At the nth sampling, the total heat energy in the furnace is ${\hat{Q}}_{n}$, the heat energy transferred to the furnace by the heater is ${∆Q}_{n}$, and the heat energy lost through the furnace surface is $∆Q_{ln}$. The heat energy in the furnace at the *n* + 1th sampling is given by Equation (1).

(1) $${\hat{Q}}_{n+1}={\hat{Q}}_{n}+{∆Q}_{n}-∆Q_{ln}\;\;\;\;\;$$

The heat capacity of the furnace is *C*, the furnace temperature is $T_{n}$, the sampling time is ∆T, the thermal conductivity coefficient of the furnace surface is *k*, and the ambient temperature is $T_{e}$. ${\hat{Q}}_{n}$ and $∆Q_{ln}$ are expressed by Equations (2) and (3), respectively.

(2) $${\hat{Q}}_{n}=C\cdot T_{n}$$

(3) $$∆Q_{ln}=k(T_{n}-T_{e})∆T\;\;\;$$

Introducing the furnace temperature decay coefficient $\rho =1-\frac{k\cdot ∆T\;}{C}$, the temperature increment $∆T_{n}=\frac{∆Q_{n}}{C}$ provided by the heater, and the ambient temperature compensation coefficient $k_{e}=\frac{k\cdot ∆T\;}{C}=1-\rho$, Equation (1) can be further expressed as Equation (4).

(4) $$T_{n+1}=\rho \cdot T_{n}+{∆T}_{n}+k_{e}\cdot T_{e}\;$$

Typically, the furnace body is well insulated, ρ→1, $k_{e}\approx 0$, and the furnace temperature is much higher than the ambient temperature, $T_{n}\gg T_{e}$, so the ambient temperature compensation term $k_{e}\cdot T_{e}$ can be ignored, and Equation (4) is simplified to Equation (5).

(5) $$T_{n+1}=\rho \cdot T_{n}+{∆T}_{n}\;\;\;\;\;\;$$

Due to signal transmission, heater response, and thermal conduction lag, heat is not immediately transferred to the furnace. At the nth sampling, the heater control signal is $C_{n}$, the heat generated by the heater is $Q_{n}$, the sampling window size is *N*, and the heat transfer process from the heater to the furnace is expressed by Equation (6).

(6) $${∆T}_{n}=w^{t}{\cdot Q}_{n}[N]\;\;\;$$

where $Q_{n}[N]=[Q_{n},\;Q_{n-1},\;\ldots ,\;Q_{n-N+1}]$, w is an *N* × 1 lag weight vector satisfying the constraints $w_{i}\geq 0,\;\forall i\in [0,\;N)$, and $\sum_{i=0}^{N-1}w_{i}=1$. Combining Equations (5) and (6), we obtain the discrete model (7) for furnace temperature.

(7) $$T_{n+1}=\rho \cdot T_{n}+w^{t}{\cdot Q}_{n}[N]$$

where ρ is the damping coefficient, which characterizes the natural decay rate of the furnace temperature in the absence of external heating. It reflects the system’s thermal inertia and insulation performance. $w^{t}$ is the lag weight vector, which describes the distributed delay of heat transfer from the heater to the furnace over multiple sampling intervals, capturing the thermal conduction lag inherent to the system.

When w is a single-point activation vector (${w=w_{\tau }\cdot e}_{n}(n_{\tau })$), Equation (7) degenerates into Equation (8).

(8) $$T_{n+1}=\rho \cdot T_{n}+w_{\tau }{\cdot Q}_{n-n_{\tau }}$$

Many researchers [1,8,14,15,16,17,20,23,25,28] have modelled the electric heating process as a first-order inertial process with time delay (Equation (9)).

(9) $$G(s)=\frac{K}{Ts+1}e^{-\tau s}$$

Equation (9) can be transformed by Laplace inversion to obtain the time domain expression (10).

(10) $$T\cdot \frac{dT(t)}{dt}+T(t)=K\cdot Q(t-\tau )\;$$

The time discretization of Equation (10) is performed so that the sampling period is ∆t, the sampling time t=n∆t, $T_{n}=T(n∆t)$, $Q_{n}=Q(n∆t)$, and the number of delayed sampling points $n_{\tau }=\tau /∆t$, and the approximate derivatives using the forward difference method, ${\left.\frac{dT(t)}{dt}\right|}_{t=n∆t}\approx \frac{T_{n+1}-T_{n}}{∆t}$, are collapsed to Equation (11).

(11) $$T_{n+1}=(1-\frac{∆t}{T})\cdot T_{n}+\frac{K\cdot ∆t}{T}\cdot Q_{n-n_{\tau }}$$

Comparing with Equation (8), the damping coefficient is given by $\rho =1-\frac{∆t}{T}$ and the gain coefficient is $w_{\tau }=\frac{K\cdot ∆t}{T}$. Therefore, Equation (7) is a generalization of Equation (9). Using Equation (7) as the mathematical model for the electric heating furnace system enables a more precise description of the electric heating process. The following section presents the identification methods for the model parameters ρ and *w*.

#### 2.1.2. Model Identification

Typically, model identification requires the prior acquisition of the open-loop step response curve of the process object [1], followed by the calculation of model parameters based on the curve. The calculated model parameters are only used for parameter tuning of PID and other control algorithms in the simulation environment, and remain unchanged during control algorithm operation, i.e., static modelling. This paper uses Equation (7) as the system model, which has up to *N* + 1 parameters. Static modelling methods struggle to maintain precise identification of the system in complex real-world environments over the long term. Therefore, it is necessary to adjust the model parameters in real time while the control algorithm is running, i.e., dynamic modelling.

This paper uses an auto-encoder for unsupervised learning of model parameters. Based on the current system model parameters and historical furnace temperature data, the furnace temperature data for the past *N* sampling points is reconstructed. The system model parameters are then adjusted based on the reconstruction error. The specific adjustment method is as follows.

According to Equation (7), at the nth sampling point, the prior estimate of the furnace temperature is given by Equation (12), and the initial conditions are given by Equation (13).

(12) $${\hat{T}}_{n}=\rho \cdot {\hat{T}}_{n-1}+w^{t}{\cdot Q}_{n-1}[N]$$

(13) $${\hat{T}}_{n-N+1}=\rho \cdot T_{n-N}+w^{t}{\cdot Q}_{n-N}[N]$$

Combining Equations (12) and (13) yields the furnace temperature reconstruction Equation (14).

(14) $$\left[\begin{array}{ccc} \begin{array}{ccc} 1 & -\rho & 0\\ 0 & 1 & -\rho \end{array} & \ldots & \begin{array}{c} 0\\ 0 \end{array}\\ \vdots & \ddots & \vdots \\ \begin{array}{ccc} 0 & 0 & 0 \end{array} & \ldots & 1 \end{array}\right]\left[\begin{array}{c} {\hat{T}}_{n}\\ \begin{array}{c} {\hat{T}}_{n-1}\\ \vdots \end{array}\\ {\hat{T}}_{n-N+1} \end{array}\right]=\left[\begin{array}{ccc} \begin{array}{cc} Q_{n-1} & Q_{n-2}\\ Q_{n-2} & Q_{n-3} \end{array} & \ldots & \begin{array}{c} Q_{n-N}\\ Q_{n-N-1} \end{array}\\ \vdots & \ddots & \vdots \\ \begin{array}{cc} Q_{n-N} & Q_{n-N-1} \end{array} & \ldots & Q_{n-2N} \end{array}\right]\left[\begin{array}{c} w_{0}\\ \begin{array}{c} w_{1}\\ \vdots \end{array}\\ w_{N-1} \end{array}\right]+\left[\begin{array}{c} 0\\ \begin{array}{c} 0\\ \vdots \end{array}\\ \rho \cdot T_{n-N} \end{array}\right]\;U\cdot \hat{T_{n}}=Q_{n}\cdot w+\rho \cdot T_{n-N}\cdot e_{N}$$

The reconstruction error is $\varepsilon_{n}=\hat{T_{n}}-T_{n}$, and the prior error is measured using the squared loss $L_{n}={\varepsilon_{n}}^{t}\varepsilon_{n}$, $\delta_{\hat{T}}=\frac{\partial L_{n}}{\partial \hat{T_{n}}}$. According to the chain rule, the gradient of the loss with respect to the model parameters is given by Equations (15) and (16).

(15) $$\delta_{w}=\frac{\partial L_{n}}{\partial w}={\left(U^{-1}\cdot Q_{n}\right)}^{t}\;\delta_{\hat{T}}$$

(16) $$\delta_{\rho }=\frac{\partial L_{n}}{\partial \rho }={\delta_{\hat{T}}}^{t}\left(\frac{\partial U^{-1}}{\partial \rho }\cdot U\cdot \hat{T_{n}}+U^{-1}\cdot T_{n-N}\cdot e_{n}\right)\;=\sum_{i=0}^{N-2}{\delta_{{\hat{T}}_{n-i}}\cdot \hat{T}}_{n-i-1}+{\delta_{{\hat{T}}_{n-N-1}}\cdot T}_{n-N}$$

Considering that the model parameters ρ and w are subject to constraints, directly truncating ρ to the interval [0, 1] and truncating $w_{i}$ to [0, +∞) will result in truncation errors. Furthermore, truncating *w* will also lead to energy non-conservation ($\sum_{i=0}^{N-1}w_{i}\neq 1$). Therefore, the mapping method shown in Equations (17) and (18) is adopted for the model parameters.

(17) $$\rho =1-LeakyExp(\tilde{\rho })=1-\left\{\begin{array}{c} e^{k_{\rho }\cdot \tilde{\rho }+\rho_{0}},\tilde{\rho }<0\\ \gamma \cdot \tilde{\rho }+e^{\rho_{0}},\tilde{\rho }>0 \end{array}\right.$$

(18) $$w_{i}=Softmax(\tilde{w_{i}})=\frac{e^{\tilde{w_{i}}}}{\sum_{i=0}^{N-1}e^{\tilde{w_{i}}}}$$

where $k_{\rho }$ is the decay rate growth coefficient, γ is the decay rate leakage coefficient, and $\rho_{0}$ is the decay rate leakage threshold. After parameter mapping, the gradient of the loss with respect to the model parameters is expressed as Equations (19) and (20).

(19) $$\delta_{\tilde{w}}=\frac{\partial L_{n}}{\partial w}\cdot \frac{\partial w}{\partial \tilde{w}}=(\delta_{w}-w^{t}\cdot \delta_{w})⊙w\;$$

(20) $$\delta_{\tilde{\rho }}=\frac{\partial L_{n}}{\partial \rho }\cdot \frac{\partial \rho }{\partial \tilde{\rho }}=\delta_{\rho }\cdot \left\{\begin{array}{c} k_{\rho }(1-\rho ),\tilde{\rho }<0\\ \gamma ,\tilde{\rho }>0 \end{array}\right.$$

In summary, the identification process for the system model parameters is as follows: first, reconstruct the furnace temperature according to Equation (14) and calculate the reconstruction error; then, use the gradient descent method according to Equations (19) and (20) to update the mapped furnace temperature decay coefficient $\tilde{\rho }$ and lag weight vector $\tilde{w}$.

### 2.2. Design of Electric Heating Control Algorithm

The large time delay in the electric heating process causes the control signal to have lag, which can easily lead to time delay overshoot problems. At the same time, the nonlinearity and time-varying nature of the electric heating process place higher demands on the predictive ability, adaptive ability, and robustness of the control algorithm.

Auto-encoder technology can learn system characteristics based on the reconstruction error of the furnace temperature, accurately model the controlled object in real time, and predict the future furnace temperature, which is expected to alleviate time delay overshoot, reduce model accuracy dependence, and enhance the adaptability of the controller. Fuzzy control technology introduces expert experience to analyze the state of the controlled object and automatically adjust the control parameters. The use of fuzzy controllers to adjust control parameters in real time is expected to further reduce the dependence of the control effect on model accuracy and improve the robustness of the control algorithm.

Combining the advantages of auto-encoder technology and fuzzy control technology, this paper proposes a composite control method—fuzzy PI control with auto-encoder predictive compensation—for dynamically constructing system models, predicting future temperatures in real time, and accurately responding to target temperatures. This chapter introduces the specific control method.

#### 2.2.1. Logical Structure of the Controller

In traditional PID control, the role of the D controller is to predict the future trend of the system through the rate of change of the error, so as to make adjustments in advance and reduce overshoot and oscillation phenomena. However, the D controller is very sensitive to noise and is prone to control instability under disturbances. This paper introduces Auto-Encoder Prediction Compensation to replace the D controller, and uses the model parameter method to predict the control amount in order to make a more accurate estimate of the future system state. Auto-encoder predictive compensation not only reduces sensitivity to noise, but also better adapts to complex system characteristics such as nonlinearity and time variability. On this basis, this paper introduces a fuzzy logic controller to adjust the PI parameters in real time, increasing the robustness of the control. This paper refers to the above control method as fuzzy PI control with auto-encoder predictive compensation, and the controller using this method is called a fuzzy PI controller with auto-encoder predictive compensation.

**Figure 1:**
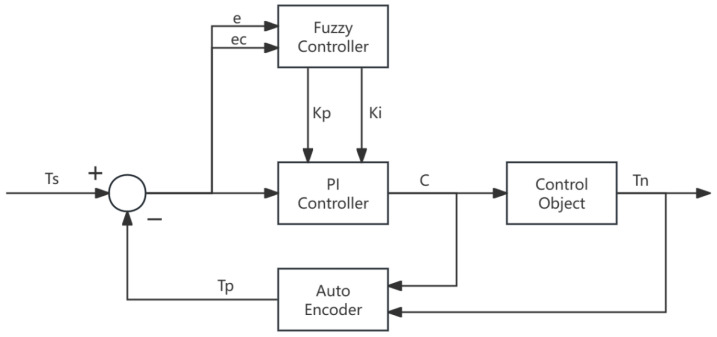
Logical structure of fuzzy PI controller with AE predictor compensation.

The fuzzy PI controller with auto-encoder predictive compensation is an intelligent temperature controller with dynamic modelling, long-term predictive feedback, and adaptive control parameter adjustment. It consists of an auto-encoder, a fuzzy PI parameter controller, and a PI controller. The overall structure of the controller is shown in Figure 1.

The PI controller generates control signals C based on the temperature error e and its derivative ec. The PI control parameters $K_{p}$ and $K_{i}$ are determined by the fuzzy controller (see Section 2.2.3). The auto-encoder (see Section 2.2.2) is located in the feedback loop, responsible for modelling the system dynamics and predicting the future temperature $T_{p}$. $T_{p}$ serves as the feedback input for the PI controller, forming a closed-loop control system.

#### 2.2.2. Auto-Encoder Design

**Figure 2:**
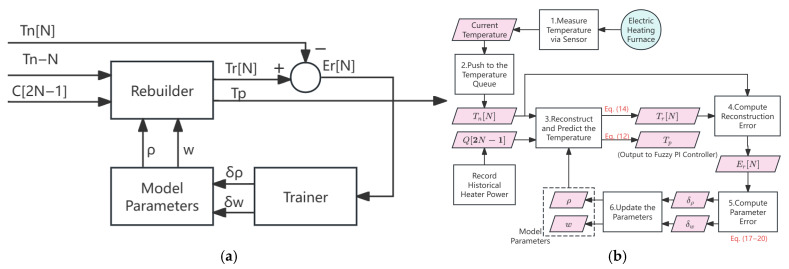
Logical structure and training process of dynamic modeller and predictor based on auto-encoder: (**a**) logical structure; (**b**) training process.

The auto-encoder consists of model parameters, a reconstructor (predictor), and a trainer, which are used for unsupervised learning of effective representations of furnace temperature data. The structure and training process of the auto-encoder are shown in Figure 2.

In Figure 2a, the model parameters include the furnace temperature decay rate ρ and the lag weight vector *w*(*N*); see Section 2.1.1 for details. The reconstructor is responsible for reconstructing the historical temperatures and predicting the future temperatures, and then inputs the prediction error $E_{r}$ to the trainer, which is used to adjust the model parameters.

The auto-encoder-based prediction model is trained online in each sawmpling frame, following a structured process as shown in Figure 2b. The entire process consists of three main stages, as detailed below:

1. Temperature Acquisition and Enqueueing: The current furnace temperature is measured via sensors and pushed into a historical temperature queue that maintains the most recent temperature samples. At the same time, the historical power input to the heater is recorded to support accurate temperature reconstruction and model training.
2. Temperature Reconstruction and Prediction: The reconstructor uses the furnace temperature reconstruction equation (Equation (14)) to calculate reconstructed temperatures for the most recent *N* sampling points. The difference between the reconstructed temperature and the true measured temperature gives the reconstruction error. Based on this reconstructed state, the auto-encoder model performs M-step iterative prediction (Equation (12)) to estimate the future furnace temperature, which is then forwarded to the fuzzy PI controller for control decision making.
3. Error Computation and Parameter Update: The trainer computes the parameter error based on Equations (17)–(20), which quantify the deviation between the predicted behaviour and actual measurements. These errors are backpropagated to update the internal model parameters using gradient descent. This online training ensures that the model can adapt to evolving furnace conditions.

Through this closed-loop mechanism, the auto-encoder not only reconstructs historical furnace dynamics but also continuously learns from real-time deviations, thereby enhancing its generalization and predictive accuracy under varying operating conditions.

#### 2.2.3. Fuzzy PI Parameter Controller Design

**Figure 3:**
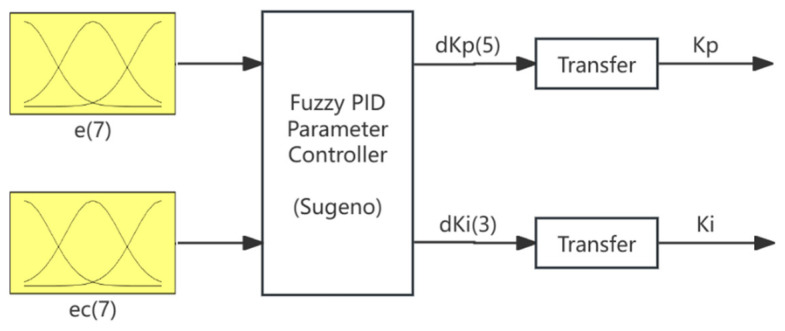
Fuzzy PI parameter controller.

The fuzzy PI parameter controller is used to control the parameters $K_{p}$ and $K_{i}$. The temperature error e and the differential error ec are used as inputs to the fuzzy PI parameter controller. According to the rules of fuzzy control, through the processes of fuzzification, fuzzy operations, and defuzzification, real-time adjustment of the PID control parameters can be achieved. The structure of the fuzzy PI parameter controller is shown in Figure 3.

Although adaptive fuzzy control methods have been widely studied as modern alternatives, they often suffer from increased system complexity and reduced interpretability due to online tuning mechanisms. In contrast, manually designed rule bases offer greater transparency and ease of implementation, especially in systems requiring strict safety margins and engineering interpretability. To reduce the computational requirements for deploying the controller, a Sugeno-type fuzzy controller with manually crafted rules is used. The domain range for the temperature error is defined as [−50, 50], and the domain range for the error difference is defined as [−6, 6]. The fuzzy sets for the temperature error and difference are {NB, NM, NS, Z, PS, PM, PB}. The domain range for the control parameter increments $dk_{p}$ and $dk_{i}$ is defined as [−6, 6]. The fuzzy sets for $dk_{p}$ are {NB, NS, Z, PS, PB}, with a range of {−6, −3, 0, 3, 6}; the fuzzy sets for $dk_{i}$ are {N, Z, P}, with a range of {−6, 0, 6}.

**Figure 4:**
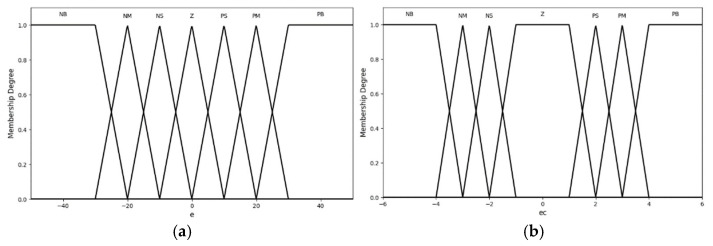
Membership functions: (**a**) error; (**b**) difference in error.

After determining the fuzzy sets and domains for each input fuzzy variable, membership functions were established for the fuzzy sets. The membership functions are triangular membership functions (trimf) and trapezoidal membership functions (trapmf). The membership functions for the input variables are shown in Figure 4.

Based on the temperature error e and the differential error ec, the fuzzy controller can adjust the parameters $k_{p}$ and $k_{i}$ in real time. We establish corresponding fuzzy rule tables for ${dk}_{p}$ and ${dk}_{i}$, as shown in Table 1 and Table 2.

**Figure 5:**
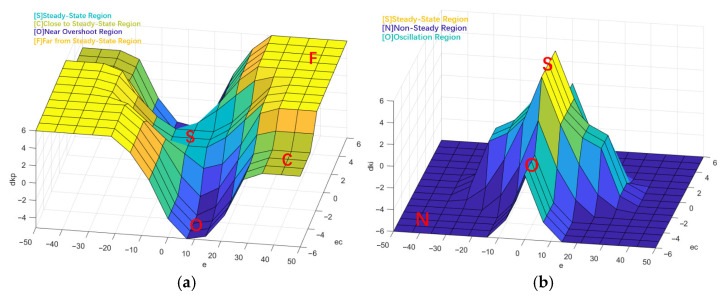
Rule surface of fuzzy PI parameter controller: (**a**) $dk_{p}$; (**b**) $dk_{i}$.

Based on the fuzzy rules and value domains of $dk_{p}$ and $dk_{i}$, the rule surface is drawn, as shown in Figure 5.

The fuzzy rule surface of ${dk}_{p}$ is divided into a steady-state region, close-to-steady-state region, near-overshoot region, and far-from-steady-state region. The far-from-steady-state region represents an interval with large error and a continuing increase trend. ${dk}_{p}$ is increased to PB to accelerate the regulation speed. The close-to-steady-state region represents an interval with large error but a decreasing trend. ${dk}_{p}$ is appropriately increased to PS to prevent overshoot. The near-overshoot region represents an interval with small errors and a tendency toward overshoot. In this case, ${dk}_{p}$ is decreased to prevent overshoot; the steady-state region represents an interval with small errors and small error changes. The original $k_{p}$ is maintained without modification.

The fuzzy rule surface of ${dk}_{i}$ is divided into three intervals: the steady-state region, non-steady-state region, and oscillation region. The steady-state region represents an interval with small error and low error change rate, where ${dk}_{i}$ is increased to reduce steady-state error. The non-steady-state region represents an interval with excessive error, primarily controlled by the proportional controller, so ${dk}_{i}$ is reduced. The oscillation region represents an interval with small error but a tendency toward oscillation, where ${dk}_{i}$ is appropriately increased to reduce steady-state error and prevent overshoot.

## 3. Results and Discussion

**Figure 6:**
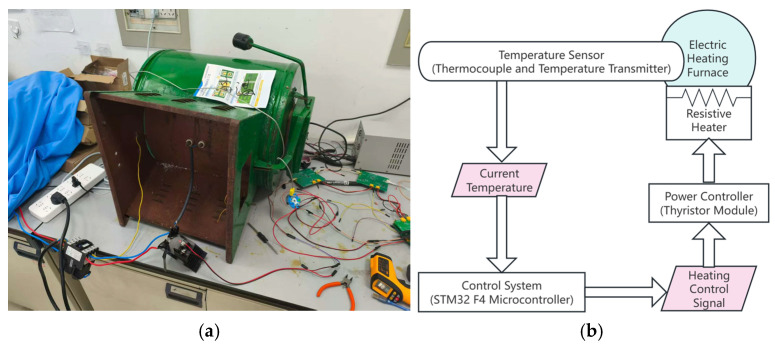
Experimental muffle furnace and system structure: (**a**) the custom-built muffle furnace used for temperature control experiments; (**b**) structural schematic of the furnace system.

To evaluate the effectiveness and stability of the proposed control method, and to assess the overall performance of the system, simulation analysis is conducted to provide reliable data support and optimization guidance for practical implementation. The simulation parameters are derived from the custom-built muffle furnace shown in Figure 6. Temperature measurements are obtained using a K-type thermocouple (WRNK-191, Kaipusen Technology Industrial Co., Ltd., Guangzhou, China) with a sampling interval of 1 s. The main sources of noise are considered to include environmental disturbances, sensor measurement errors, and fluctuations in the power supply.

### 3.1. Model Estimation

**Figure 7:**
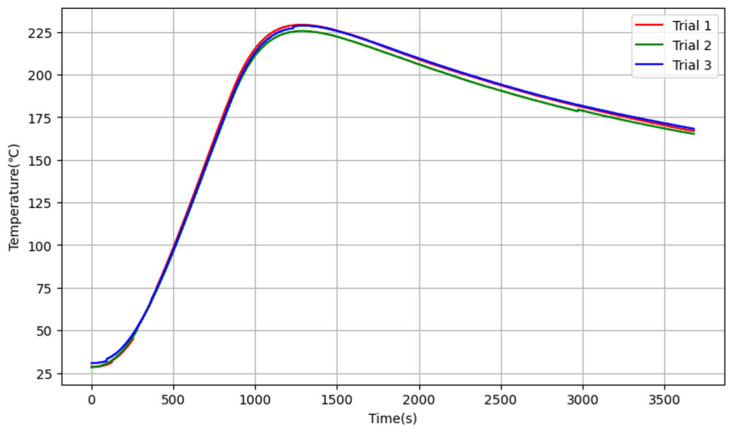
The open-loop response of the electric heating furnace.

An open-loop control test was conducted on the custom-built muffle furnace shown in Figure 6, which was developed by the research team. The system was heated at 10% of the maximum heating power for 816 s, after which heating was stopped, and the system was allowed to cool naturally. The results of three independent tests of the system’s open-loop response curve are shown in Figure 7.

For convenience of calculation, a simplified model shown in Equation (9) is used for parameter estimation. The time interval 1309–3680 s is the temperature decay phase, and the furnace temperature decay coefficient $\rho =\frac{E[T(n)T(n-1)]}{E[{T(n-1)}^{2}]}=0.99987$ is calculated using the data from this time interval. The time interval 258–900 s is the linear heating phase, the linear heating rate $\dot{T}=\frac{E\left[\left(T-E\left[T\right]\right)\left(t-E\left[t\right]\right)\right]}{E\left[{\left(t-E\left[t\right]\right)}^{2}\right]}=0.23984\;({}^{\circ}C/s)$, and the single-point hysteresis weight $w_{\tau }=\frac{\dot{T}}{C_{10\%}}=0.1499\;({}^{\circ}C/s\cdot mA)$. The time delay from system start-up to start of heating is $\tau_{1}=42\;s$, and the time delay from system shutdown to stop of heating is $\tau_{2}=444\;s$. The average of the two is calculated to obtain the estimated system delay $\tau \approx \frac{\tau_{1}+\tau_{2}}{2}=243\;s$. Using the impulse response invariance method, the estimated values of the system transfer function parameters are *T* = 7692 s, *K* = 1153. Therefore, the transfer function of the electric furnace system can be approximately represented by Equation (21).

(21) $$G(s)=\frac{1153}{7692\;s+1}e^{-243s}\;\;$$

### 3.2. Dynamic Modelling Simulation

**Figure 8:**
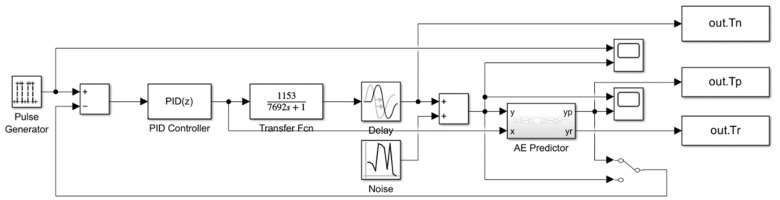
Pre-training and dynamic modelling simulation ($K_{p}=0.02,\;K_{i}=0.000001$).

To verify the dynamic modelling capabilities of the auto-encoder, dynamic modelling simulations are required. Prior to this, pre-training simulations are conducted to optimize the initial modelling and prediction performance of the auto-encoder. During pre-training simulation, the measured temperature is used as feedback; during dynamic modelling simulation, the temperature predicted by the auto-encoder is used as feedback. The simulation structure is shown in Figure 8. Due to the complexity of the auto-encoder’s implementation logic, C++ code is written using MATLAB/Simulink (R2023b)’s S-Function module.

Considering that the accuracy of commonly used thermocouple temperature transmitters (e.g., PT100) is typically 0.2%, the measurement accuracy at a range of 1000 °C is 2 °C, and the measurement noise is set to Gaussian noise with a standard deviation of 2 °C. Additionally, the sampling time of the automatic encoder is set to 10 s to obtain a longer prediction time.

**Figure 9:**
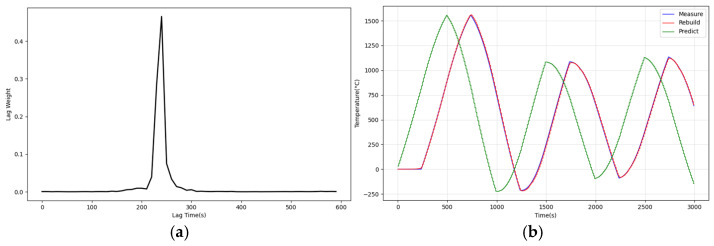
Pre-training simulation results: (**a**) parameter estimation of lag weight; (**b**) measured, reconstructed, and predicted temperature.

The pre-training simulation uses a square wave with an amplitude of 1000 °C, a period of 1000 s, and a duty cycle of 50% as the excitation signal. After the pre-training simulation, a 1000 °C step excitation is used to verify the pre-training effect. Figure 9 shows the results of the pre-training simulation.

According to Figure 9a, the Auto-Encoder’s estimate of the lagged weight vector is approximately equal to the impulse at a lag time of 243 s, which is consistent with the system model used in the simulation. According to Figure 9b, the reconstruction error is only 13.1 °C, and the prediction error is only 7.8 °C (root-mean-square error). Therefore, we believe that the auto-encoder can accurately model the system with small reconstruction and prediction errors, providing accurate temperature predictions.

**Figure 10:**
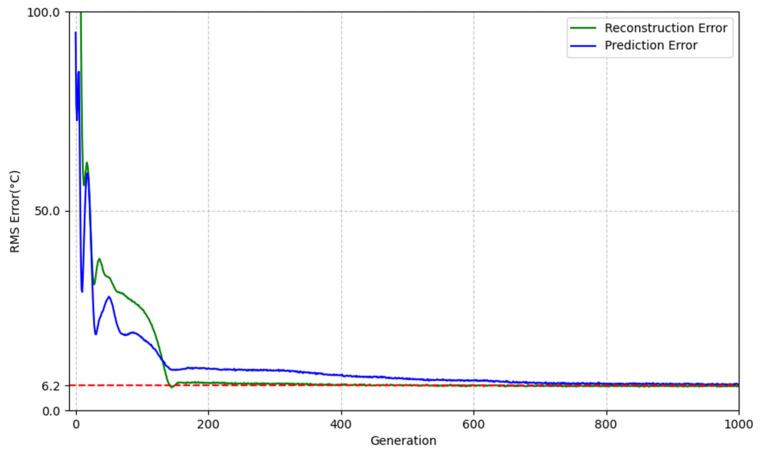
Learning curve of the dynamic modelling process.

**Figure 11:**
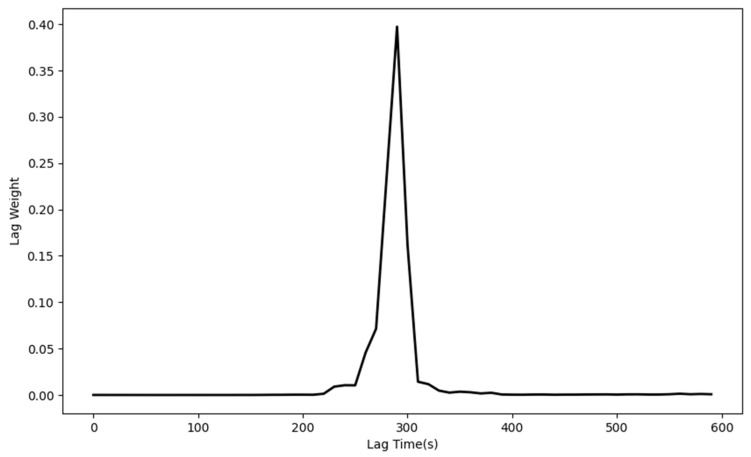
Parameter estimation of lag weight vector (after dynamic modelling).

The above results show that the auto-encoder has good static performance. In order to examine the modelling and prediction capabilities of the auto-encoder in time-varying systems, we modified the model parameters to test its dynamic performance. The model was initialized using pre-trained parameters, and a square wave with an amplitude of 1000 °C, a period of 1000 s, and a duty cycle of 50% was again used as the excitation signal. Considering that system ageing may cause reduced system gain due to oxidation of the heating elements, increased system inertia due to ageing of the housing and insulation materials, and increased system delay due to ageing of the controller or sensors, the model parameters were modified to *T* = 9230 s (+20%), *K* = 922 (−20%), and τ = 292 s (+20%) to simulate the time variation of the system and perform dynamic modelling simulation. Figure 10 shows the learning curve of the dynamic modelling process, and Figure 11 shows the model parameter estimation results of the Auto-Encoder after dynamic modelling simulation.

According to Figure 10, the reconstruction error and prediction error rapidly decreased to about 10 °C within 148 generations and stabilized at 6.2 °C after 810 generations. According to Figure 11, the Auto-Encoder’s estimate of the lagged weight vector is approximately equal to the impulse at a lag time of 292 s, which is consistent with the system model used in the simulation. Therefore, we believe that the Auto-Encoder is capable of accurately modelling dynamics by following changes in system model parameters.

### 3.3. Control System Step Simulation

**Figure 12:**
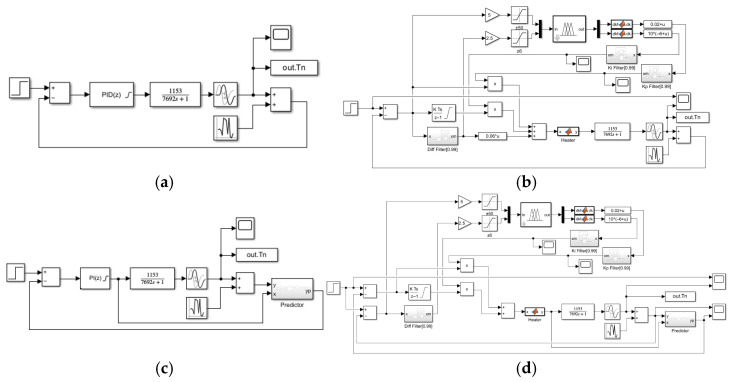
Step response simulation structure: (**a**) PID control ($K_{p}=0.01\sim 0.02,\;K_{i}=0.000001,\;K_{d}=0.06)$; (**b**) fuzzy PID control ($K_{p}=0.01\sim 0.03,\;K_{i}=10^{-7}\sim \;2\cdot 10^{-5},\;K_{d}=0.06)$; (**c**) PI control with AE Prediction Compensation ($K_{p}=0.03,\;K_{i}=0.000001)$; and (**d**) fuzzy PI control with AE Prediction Compensation ($K_{p}=0.01\sim 0.05,\;K_{i}=10^{-7}\sim \;2\cdot 10^{-5})$.

In order to examine the control accuracy and response speed of the system, we performed system step simulations of PID control, fuzzy PID control, PI control with Auto-Encoder predictive compensation, and fuzzy PI control with Auto-Encoder predictive compensation. The simulation structure is shown in Figure 12.

**Figure 13:**
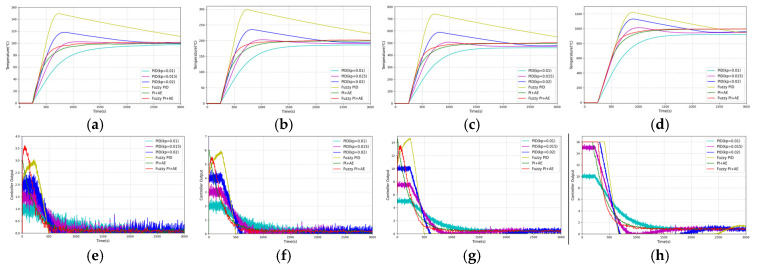
Control system step responses and actuator curves at different target temperatures: step responses: (**a**) 100 °C, (**b**) 200 °C, (**c**) 500 °C, and (**d**) 1000 °C; actuator curves: (**e**) 100 °C, (**f**) 200 °C, (**g**) 500 °C, and (**h**) 1000 °C.

As in Figure 12, we use a step signal as the excitation and assume that the heater has good linearity. Considering the effective input range of the heater, the control variable is truncated to [0, 16]. To ensure the stability of the fuzzy controller’s input and output, exponential moving average filters are added to its output and differential input terminals. The step responses and actuator curves at target temperatures of 100 °C, 200 °C, 500 °C, and 1000 °C are shown in Figure 13, and the performance metrics are listed in Table 3.

According to Figure 13 and Table 3, for a nonlinear, unilateral temperature control system with a large time delay, the PID control effectiveness is significantly affected by the $K_{p}$: a low $K_{p}$(0.01) results in the inability to enter the error band at the observation time; a medium $K_{p}$(0.015) provides a faster rise time (about 430 s) and a lower amount of overshoot (1.3~2.4%); and a higher $K_{p}$(0.02) achieves the fastest rise time (282~350 s) but results in significant overshoot (13.1~17.8%) and longer settle time (1710~2518 s). Meanwhile, the PID control shows significant changes in the actuator curve shape at different target temperatures and fails to even enter the error band within the observation time in the temperature range of 500 to 1000 degrees Celsius, indicating its inability to adapt to the nonlinear characteristics of the system. Moreover, the ITAE value of PID ($K_{p}$ = 0.015) is the lowest among the three groups of PID control from 100 °C to 500 °C, but when the temperature reaches 1000 °C, the ITAE of PID ($K_{p}$ = 0.02) is instead the lowest, which indicates that the stability of PID control is significantly correlated with the target temperature, and that the optimality of the parameter selection changes with the target temperature. In addition, the lack of predictive compensation of the PID control leads to a lag in the temperature feedback, which results in an over-response of the control output and prolongs the regulation time as evidenced by the large width of its actuator curve peaks and a high level of the controller output even when approaching the overshoot. These problems indicate that PID control has difficulty in balancing speed, stability, and robustness in this system, and that parameter tuning requires a trade-off between speed, amount of overshoot, and stabilization time.

The fuzzy PID control reduced the rise time by 21% compared to the PID control ($K_{p}$ = 0.02), but increased the amount of overshoot by a factor of 1.6 due to the lack of predictive compensation. The PI + AE control uses the predicted value as feedback, eliminating the overshoot caused by the time delay, and its actuator curve has the smallest peak width and falls steeply, but the rise time is 78% longer than that of the PID control ($K_{p}$ = 0.02) due to the premature decay of the control signal. The fuzzy PI + AE control retains the advantage of the very low overshoot amount of the PI + AE control (0.0~1.3%), while at the same time, the rise time is 29% shorter compared with that of the PI + AE control, the regulation time is 19% shorter, and it has the lowest ITAE. The actuator curve of the fuzzy PI + AE control has moderate peak widths, rapid descents, gentle decays, and consistent curve shapes when the actuators are not saturated, which show nonlinear adaptability and are the best performers among all the evaluated controllers.

Therefore, the fuzzy PI control with auto-encoder predictive compensation proposed in this paper combines the advantages of fuzzy control and the auto-encoder. Simulation results suggest that it helps mitigate time-lag-induced overshoot and improve control speed, and shows promising performance in achieving accurate and stable temperature regulation under simulation conditions.

### 3.4. Disturbance-Resistant Simulation

**Figure 14:**
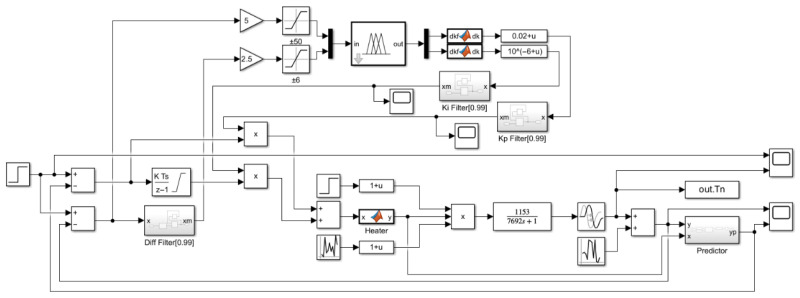
Step response simulation in complex disturbance environment.

The operating environment of an electric heating furnace is complex and variable, and the control system may be affected by various disturbances such as measurement errors, external temperature fluctuations, and power grid voltage fluctuations. Therefore, it is necessary to conduct disturbance-resistant simulations to evaluate the controller’s performance under complex disturbance conditions, verify its robustness and reliability, and ensure the system’s stability and control accuracy during actual operation. The structure of the disturbance-resistant simulation is shown in Figure 14.

**Figure 15:**
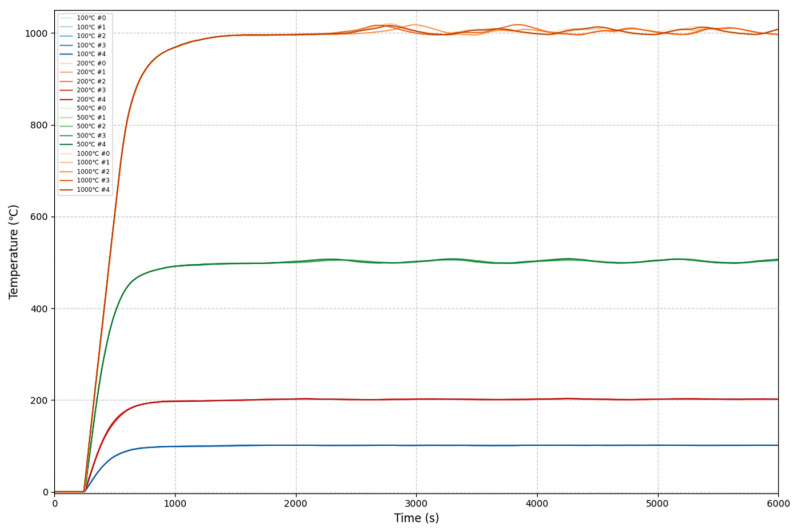
Step response in complex disturbance environment.

The simulation environment shown in Figure 14 takes into account Gaussian measurement noise with a standard deviation of 2 °C. It also considers grid voltage fluctuations by superimposing ±10% uniformly distributed noise (power supply voltage deviation limit [34]) on the controller’s control signal output to simulate the impact of unstable power supply voltage on the control. To account for sudden changes in thermal load, a 10% system gain step is added at 1500 s to simulate a sudden decrease in thermal load within the furnace (due to the single-sided controllability of the actuators in the electric heating system, a sudden decrease in thermal load can cause a sudden increase in system gain, which may lead to overshoot). Step signals of 100 °C, 200 °C, 500 °C, and 1000 °C were used as stimuli for system interference resistance simulation, and for each target temperature, five independent experiments were conducted to evaluate repeatability and robustness. The system response is shown in Figure 15, and the performance metrics are listed in Table 4.

According to Figure 15 and Table 4, the fuzzy PI controller with auto-encoder-based prediction compensation maintains the overshoot within 2% under complex disturbance conditions. Compared to the disturbance-free scenario, the rise time is reduced by 7%, the settling time is shortened by 10%, and the ITAE increases by no more than 7.2%, indicating no significant degradation in performance metrics. Therefore, the proposed control method demonstrates a certain degree of disturbance rejection capability and shows promise for achieving stable and reliable temperature regulation in complex real-world environments.

## 4. Conclusions

This paper addresses the challenge of temperature control in electric heating furnaces under nonlinear, time-varying, and large-time-delay conditions by proposing an intelligent control method that integrates Auto-Encoder modelling with fuzzy PI control. The method introduces an auto-encoder to perform unsupervised learning of the system’s dynamic characteristics, enabling the construction of a unified and accurate dynamic model. By using predicted temperature to replace lagging feedback, the method suppresses overshoot and response delay caused by time delays. At the same time, the fuzzy controller dynamically adjusts PI parameters, enhancing system robustness and adaptability. Simulation tests conducted on the *MATLAB/Simulink* platform show that the method maintains temperature overshoot within 2% under various disturbances (including 2 °C measurement errors, 10% voltage fluctuations, and 10% sudden gain changes), with fast response and stable performance. These results indicate that, under the evaluated conditions, the proposed method demonstrates improved accuracy and robustness compared to tested conventional PID and fuzzy control strategies, potentially offering a viable reference for advanced controller design in similar industrial contexts.

## 5. Limitations and Future Work

The proposed Auto-Encoder-based fuzzy PI control method still has several limitations. Its performance relies on the quality and coverage of training data, which may introduce data bias and limit generalization. The encoding and decoding process, along with real-time fuzzy rule evaluation, incurs moderate computational overhead, which may affect deployment in embedded or low-resource systems. Moreover, current validation is confined to SISO systems, with no demonstration of applicability to MIMO scenarios. However, the modular structure of the proposed method makes it technically extendable to MIMO systems by replicating the encoder structure for each input–output pair and applying coordinated fuzzy regulation.

Future work will focus on

1. Experimental validation of real electric heating furnaces;
2. Comparison with advanced control strategies such as MPC and robust control;
3. Implementation of lightweight variants for real-time deployment, and extension to multivariable, fault-tolerant, and dynamically uncertain systems.

## Figures and Tables

**Table 1:** Fuzzy rules of adjustment of ${dk}_{p}$.

${dk}_{p}$		ec						
		NB	NM	NS	Z	PS	PM	PB
e	**NB**	PB	PB	PB	PB	PB	PS	PS
	**NM**	PB	PB	PB	PS	Z	Z	NS
	**NS**	PS	PS	PS	Z	NS	NB	NB
	**Z**	NS	NS	Z	Z	Z	NS	NS
	**PS**	NB	NB	NS	Z	PS	PS	PS
	**PM**	NS	Z	Z	PS	PB	PB	PB
	**PB**	PS	PS	PB	PB	PB	PB	PB

**Table 2:** Fuzzy rules of adjustment of ${dk}_{i}$.

${dk}_{i}$		ec						
		NB	NM	NS	Z	PS	PM	PB
e	**NB**	N	N	N	N	N	N	N
	**NM**	N	N	N	Z	N	N	N
	**NS**	N	N	Z	Z	Z	N	N
	**Z**	Z	Z	P	P	P	Z	Z
	**PS**	N	N	Z	Z	Z	N	N
	**PM**	N	N	N	Z	N	N	N
	**PB**	N	N	N	N	N	N	N

**Table 3:** Performance metrics of each controller.

Temperature (°C)	Controller	Rise Time ^1^ (s)	Settle Time ^2^ (s)	Overshoot (%)	ITAE ^3^ ($s^{2}\cdot {}^{\circ}C$)
100	PID ($K_{p}$ = 0.01)	1094	2063	0.0	$4.6{\times 10}^{7}$
	PID ($K_{p}$ = 0.015)	433	779	2.4	$1.6\times 10^{7}$
	PID ($K_{p}$ = 0.02)	282	2231	17.8	$3.0\times 10^{7}$
	Fuzzy PID	200	3443	49.3	$1.2\times 10^{8}$
	PI+AE	528	1061	0.0	$1.8\times 10^{7}$
	Fuzz PI+AE	357	789	1.3	$1.4\times 10^{7}$
200	PID ($K_{p}$ = 0.01)	1167	>5000	0.0	$1.1\times 10^{8}$
	PID ($K_{p}$ = 0.015)	433	779	1.4	$5.5\times 10^{7}$
	PID ($K_{p}$ = 0.02)	282	1722	17.6	$6.1\times 10^{7}$
	Fuzzy PID	200	3445	49.4	$2.4\times 10^{8}$
	PI + AE	546	1022	0.0	$3.6\times 10^{7}$
	Fuzz PI + AE	362	789	1.0	$2.8\times 10^{7}$
500	PID ($K_{p}$ = 0.01)	1183	>5000	0.0	$3.0\times 10^{8}$
	PID ($K_{p}$ = 0.015)	432	>5000	1.3	$1.6\times 10^{8}$
	PID ($K_{p}$ = 0.02)	282	1710	17.6	$1.7\times 10^{8}$
	Fuzzy PID	200	3374	48.0	$5.8\times 10^{8}$
	PI + AE	529	1012	0.0	$9.0\times 10^{7}$
	Fuzz PI + AE	375	837	0.8	$6.6\times 10^{7}$
1000	PID ($K_{p}$ = 0.01)	1184	>5000	0.0	$5.9\times 10^{8}$
	PID ($K_{p}$ = 0.015)	432	>5000	1.3	$3.3\times 10^{8}$
	PID ($K_{p}$ = 0.02)	350	2518	13.1	$3.1\times 10^{8}$
	Fuzzy PID	343	2702	22.2	$4.2\times 10^{8}$
	PI + AE	566	1060	0.0	$2.0\times 10^{8}$
	Fuzz PI + AE	452	947	0.0	$1.7\times 10^{8}$

^1^ Time from 10% to 90% of steady-state value. ^2^ Time to reach and stay within the ±5% error band. ^3^ Integral of time-weighted absolute error in 3000 s.

**Table 4:** Performance metrics in complex disturbance environment of each controller.

Temperature (°C)	Rise Time ^1^ (s)	Settle Time ^2^ (s)	Overshoot (%)	ITAE ^3^ ($s^{2}\cdot {}^{\circ}C$)
100	336 ± 6	711 ± 9	1.9 ± 0.1	$(1.4\;\pm \;0.1)\times 10^{7}$
200	339 ± 5	716 ± 6	1.6 ± 0.2	$(2.6\;\pm \;0.1)\times 10^{7}$
500	336 ± 2	742 ± 2	1.6 ± 0.1	$(6.1\;\pm \;0.1)\times 10^{7}$
1000	424 ± 1	862 ± 2	1.8 ± 0.2	$(1.6\;\pm \;0.1)\times 10^{8}$

^1,2,3^ The same as Table 3.

## Data Availability

Dataset available on request from the authors.

## Abbreviations

The following abbreviations are used in this manuscript:

PID	Proportional–Integral–Derivative Control
PI	Proportional–Integral Control
AE	Auto-Encoder
ITAE	Integral of Time-Weighted Absolute Error
